# A Comparative Study of the Marginal Fit of Endocrowns Fabricated From Three Different Computer-Aided Design/Computer-Aided Manufacturing (CAD/CAM) Ceramic Materials: An In Vitro Study

**DOI:** 10.7759/cureus.40081

**Published:** 2023-06-07

**Authors:** Esraa Attar, Shatha Alshali, Tariq Abuhaimed

**Affiliations:** 1 Department of Oral and Maxillofacial Prosthodontics, King Abdulaziz University, Jeddah, SAU; 2 Department of Restorative Dentistry, King Abdulaziz University, Jeddah, SAU

**Keywords:** ips e.max cad, vita suprinity, vita enamic, marginal fit, marginal gap, endocrown

## Abstract

Introduction: The marginal seal and adaptation are important factors for successful restoration. An inadequate marginal seal can lead to bacterial microleakage, plaque accumulation, and eventually treatment failure This in vitro study aimed to compare the marginal gap of endocrowns fabricated from three different computer-aided design/computer-aided manufacturing (CAD/CAM) ceramic materials.

Methods: Thirty extracted mandibular molars were selected for the study. Endocrown preparations were completed after root canal treatment. Teeth were divided into three groups to receive endocrowns fabricated of lithium disilicate ceramic (IPS-e.max CAD, Ivoclar Vivadent AG, Schaan, Liechtenstein), zirconia-reinforced lithium silicate ceramic (VITA Suprinity®, VITA Zahnfabrik, Bad Säckingen, Germany), and polymer-infiltrated ceramic (VITA Enamic®, VITA Zahnfabrik). The digital impressions were transferred to the design software to construct the endocrowns. The endocrowns were milled and cemented. The marginal fit was examined using a digital camera stereomicroscope at a magnification of 80X. Images were transferred to Image-J software (National Institutes of Health, Bethesda, Maryland, United States) to measure the marginal gap.

Results: One-way ANOVA showed a significant difference in the marginal gap between the different ceramic groups (P=0.006). Tukey’s Honest Significant Difference (HSD) post-hoc test showed that VITA Suprinity had significantly higher gap width values than VITA Enamic (P=0.005). No significant differences in gap width values were found between VITA Enamic and IPS e.max CAD or between VITA Suprinity and IPS e.max CAD (P>0.05).

Conclusion: The marginal gap of endocrown restorations varies with different CAD/CAM materials (zirconia-reinforced lithium silicate glass-ceramic, polymer-infiltrated hybrid ceramic, and lithium disilicate glass-ceramic), but are all within clinically acceptable marginal gap width.

## Introduction

The survivability of endodontically treated teeth depends on the type and quality of the post-endodontic restoration [1-4]. Previous studies have shown that restoration of endodontically treated teeth with crowns has a survival rate similar to that of vital teeth (94.2% and 95%, respectively) [5]. Traditionally, badly destructed teeth were restored using posts and cores with full-coverage crowns [6,7]. Coronal coverage of endodontically treated teeth is crucial for the treatment's success [8]. A satisfactory coronal seal can increase the success rate of endodontic treatment from 44% to 91% [9,10].

Recently, endocrown restoration has become more popular, as it has shown long-term survival and success rates [11-13]. The popularity of this type of restoration has increased in the past decade owing to advancements in both adhesive and restorative materials [14,15]. This restoration design allows minimal tooth preparation and preservation of the remaining tooth structure. It relies on the pulp chamber and bonding material for retention. This restoration was introduced in 1995 by Pissis [16] and was named endocrown in 1999 by Bindl and Mormann [17], and they described it as a monoblock ceramic crown bonded to a depulped posterior tooth. The materials available for endocrown fabrication include zirconia-reinforced lithium silicate ceramics, fiber composites, lithium disilicate ceramics, and hybrid nanoceramics [18-20].

The marginal seal and adaptation are important factors for successful restoration [21,22]. An inadequate marginal seal can lead to bacterial microleakage, plaque accumulation, and eventually treatment failure [21]. The marginal gap can be defined as the perpendicular measurement from the internal surface of the casting to the axial wall of the preparation at the marginal level [23]. 

The aim of this in vitro study was to compare the marginal gaps of endocrown restorations fabricated using three different computer-aided design/computer-aided manufacturing (CAD/CAM) materials: zirconia-reinforced lithium silicate glass-ceramic (VITA Suprinity®, VITA Zahnfabrik, Bad Säckingen, Germany), polymer-infiltrated hybrid ceramic (VITA Enamic®, VITA Zahnfabrik), and lithium disilicate glass-ceramic (IPS e.max CAD, Ivoclar Vivadent AG, Schaan, Liechtenstein). The null hypothesis was that there is no difference in the marginal gap of endocrown restorations between the three CAD/CAM ceramic materials tested.

## Materials and methods

Thirty recently extracted mandibular molars of approximately equal sizes were selected for this study. Teeth were ultrasonically cleaned and examined to rule out cracks and fractures. The teeth were stored in a saline solution until testing. The specimens were embedded in auto-polymerizing acrylic resin (Jet XR™, Lang Dental Manufacturing Co., Inc., Wheeling, Illinois, United States) using customized molds 2 mm below the cemento-enamel junction to simulate the bone level.

A standard endodontic access cavity was prepared for all specimens. Working length was determined using a k-file of size 10 (Dentsply Sirona, Charlotte, North Carolina, United States). The teeth were instrumented using nickel-titanium (NiTi) rotary files (ProTaper Next, Dentsply Sirona) up to size X3. The canals were irrigated with sodium hypochlorite (NaOCl) solution (5.25%) after each file change. A warm vertical compaction technique was used to obturate all canals using ProTaper Next Conform Fit (Dentsply Sirona), hot condenser system B (Sybron Endo; Henry Schein, Inc., Melville, New York, United States), and B&L Beta device (B&L Biotech USA, Inc., Fairfax, Virginia, United States). Crowns were reduced by 2-3 mm, creating a 90° circumferential butt joint margin with a width of at least 2 mm using a wheel diamond bur (Brasseler USA, Savannah, Georgia, United States). A cylindrical diamond bur (Brasseler USA) with a total occlusal convergence of 7° was used to make the access cavity and coronal pulp chamber continuous by eliminating the undercuts in the access cavity. A large-diameter fine diamond bur (Brasseler USA) was used to round down the internal line angle, remove irregularities, and produce a flat polished surface. Next, after etching and bonding, a thin flowable composite resin layer was applied on the base of the pulp chamber (3M™ Filtek™ Supreme Flowable; 3M, Saint Paul, Minnesota, United States) to fill the canal orifices, flatten the floor at a depth of 3-5 mm from the occlusal surface, and minimize undercuts. The prepared teeth were then randomly divided into three groups to receive endo crowns of three different ceramic materials (Table 1).

**Table 1:** The tested materials, composition, and manufacturer

Material	Composition	Manufacturer
IPS-e.max CAD	Lithium disilicate-based ceramic containing 70% Li_2_Si_2_O_5 _crystals	Ivoclar Vivadent AG, Schaan, Liechtenstein
VITA Suprinity	Zirconia-reinforced lithium silicate ceramic containing Li_2_Si_2_O_5 _crystals and 10% ZrO_2_	Vita Zahnfabric, Bad Säckingen, Germany
VITA Enamic	Fine structure ceramic network (86%) reinforced by an acrylate polymer network (14%)	Vita Zahnfabric, Bad Säckingen, Germany

**Figure 1:**
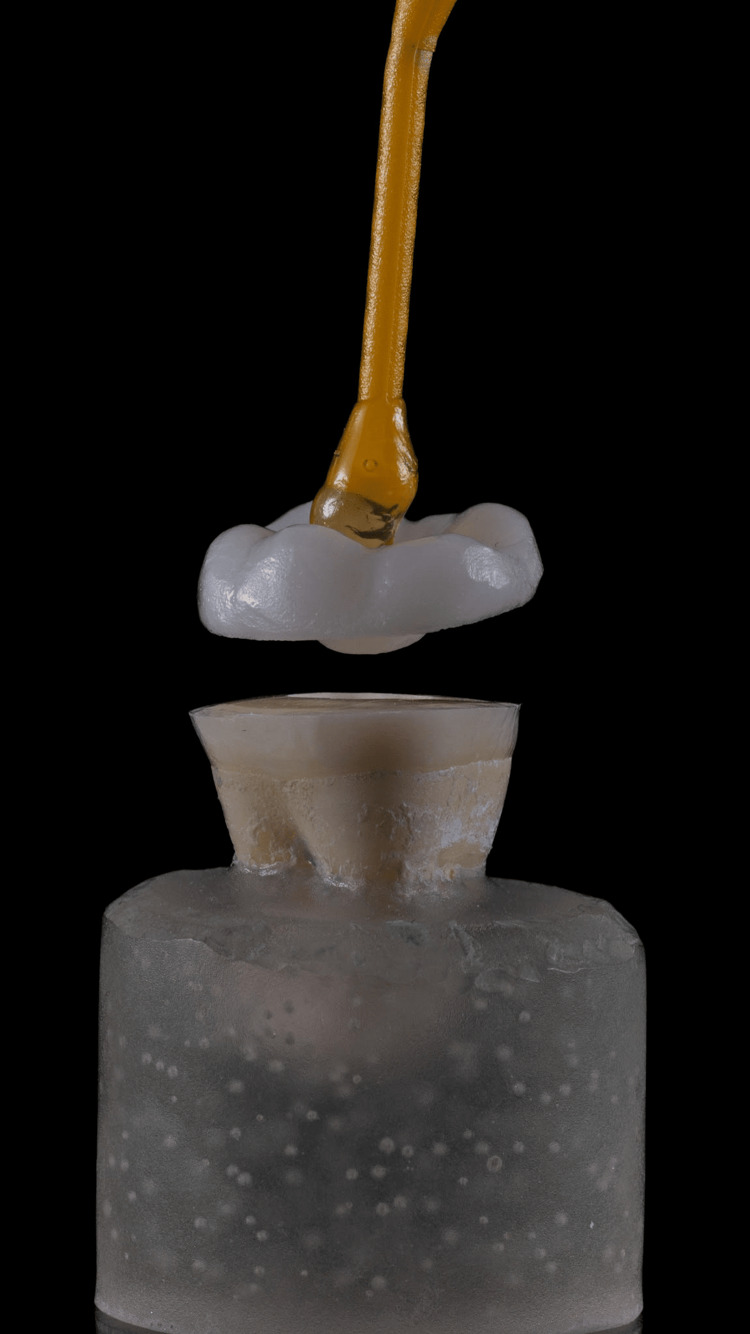
Specimen of the prepared tooth and corresponding milled endocrown

The prepared specimens were scanned using a laboratory scanner, i3Dscan (imes-icore GmbH, Eiterfeld, Germany). Digital impressions were saved as standard tesselation language (STL) files. The files were transferred to designing software (CORiTEC SmartControl; imes-icore GmbH) to construct the endocrowns. The virtual endocrowns were converted to STL files and milled under wet processing using a CORiTEC 250i Loader Pro (imes-icore GmbH). IPS-e.max CAD and VITA Suprinity samples were further crystallized using a ceramic furnace (Programat EP 5000, Ivoclar Vivadent AG) for 30 minutes and 26 minutes, respectively, to achieve their final esthetic and mechanical properties. In contrast, VITA Enamic endocrowns did not require any crystallization processes. All endocrowns were glazed and polished. All endocrowns were seated and examined for anatomy, marginal fit, and adaptation (Figure 1).

**Figure 2:**
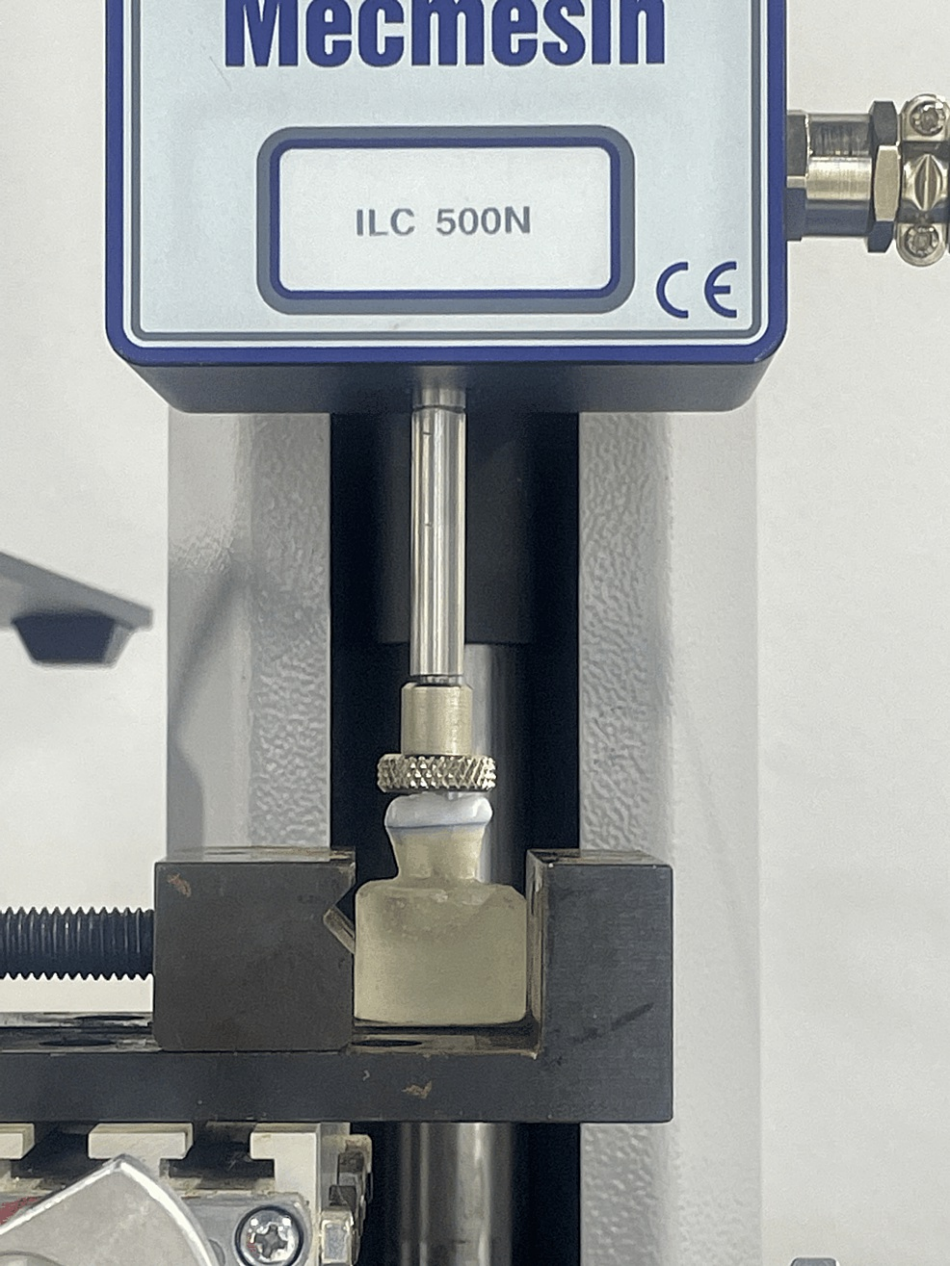
Seating of the endocrowns using a Multitest 2.5i machine* * Mecmesin Limited, Horsham, United Kingdom

The teeth were etched with 35% phosphoric acid (N-etch; Ivoclar Vivadent AG) for 15 seconds, rinsed, and dried. The endocrown’s inner surface was conditioned with 5% hydrofluoric acid for 20 seconds (HF5% Vita Ceramic Etch; Vita Zahnfabric), rinsed, and dried. A silane coupling agent (Monobond Plus; Ivoclar Vivadent AG) was applied for 60 seconds and then dried. Finally, dual-cure resin cement (3M ESPE Relyx U200 Self-Adhesive Resin Cement; 3M) was mixed with a drop of methylene blue dye and applied over the silanized surface. The endocrowns were seated using a Multitest 2.5i (Mecmesin Limited, Horsham, United Kingdom) under pressure of 50N (Figure 2). Light-curing was performed for at least 20 seconds per surface. The residual excess resin cement was removed using a microbrush and scaler. The specimens were then stored in distilled saline. A single operator performed all teeth preparations and cementation. 

**Figure 3:**
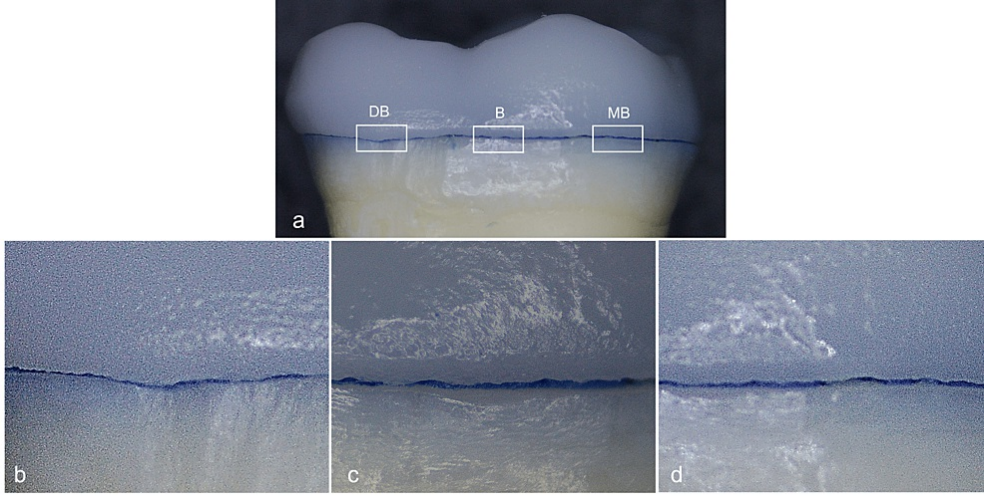
Specimen under stereomicroscope showing the midbuccal surface (B), mesiobuccal (MB), and distobuccal (DB). (a) Magnification 10x; (b, c, d) Magnification 80x

The marginal fit was examined using a digital camera stereomicroscope (ShenZhen RaySmart Technology Co., Ltd., Shenzhen, Guangdong, China) at a magnification of 80X. The specimens were positioned perpendicular to the camera by using a custom-made device. Images were recorded at eight points for each specimen as follows: buccal, mesiobuccal, mesial, mesiolingual, lingual, distolingual, distal, and distobuccal surfaces of the endocrown marginal area (Figure 3). Images were transferred to the ImageJ software (ImageJ Version 1.53t; National Institutes of Health, Bethesda, Maryland, United States) to measure the marginal gap. In Image J, all images are expressed in pixels, which require calibration using a ruler in the software to convert pixels into microns. Three measurements were recorded at each point, resulting in 24 points for each specimen. The marginal gap was measured by selecting two points to be connected with a straight line: a perpendicular line from the margin of the prepared tooth structure to the internal surface of the endocrown. 

Statistical analyses were performed using IBM SPSS Statistics for Windows, Version 23.0 (Released 2015; IBM Corp., Armonk, New York, United States). Descriptive statistics in terms of means and standard deviations were calculated for each group. One-way ANOVA was performed to compare the differences in marginal gaps between the three ceramic groups. Tukey’s Honest Significant Difference (HSD) post-hoc test was performed to compare the specific pairs of means that were significantly different. Statistical tests were conducted at a significance level of P<0.05.

## Results

The mean and standard deviation of the marginal gap values (µm) for each of the three groups are summarized in Table 2.

**Table 2:** Mean marginal gap values (µm) and standard deviations of the three tested groups IPS-e.max CAD: Ivoclar Vivadent AG, Schaan, Liechtenstein; VITA Suprinity®/VITA Enamic®: VITA Zahnfabrik, Bad Säckingen, Germany

	N.	Mean	Std. Deviation	Minimum	Maximum
VITA Suprinity	80	34.61	13.90	6.67	65.47
IPS e.max CAD	80	29.16	18.05	4.00	97.60
VITA Enamic	80	26.63	15.93	5.47	96.27

One-way ANOVA showed a significant difference in the marginal gap between the different ceramic groups (P=0.006). In other words, the material has a significant effect on the marginal gap width (Table 3).

**Table 3:** One-way ANOVA test of the mean marginal gap values of the tested ceramic materials

	Sum of Squares	df	Mean Square	F	Sig.
Between Groups	2660.461	2	1330.231	5.161	0.006
Within Groups	61081.313	237	257.727		
Total	63741.774	239			

Post-hoc Tukey’s test showed that VITA Enamic had a significantly lower marginal gap width than VITA Suprinity (P=0.005). However, there was no significant difference between VITA Enamic and IPS e.max CAD (P=0.580) or between VITA Suprinity and IPS e.max CAD (P=0.083) (Table 4).

**Table 4:** Post-hoc analysis (Tukey test) of the marginal gap for the tested ceramic materials IPS-e.max CAD: Ivoclar Vivadent AG, Schaan, Liechtenstein; VITA Suprinity®/VITA Enamic®: VITA Zahnfabrik, Bad Säckingen, Germany

(I) Type	(J) Type	Mean Difference (I-J)	Std. Error	Sig.	95% Confidence Interval	
					Lower Bound	Upper Bound
VITA Suprinity	IPS e.max CAD	5.44987	2.53834	0.083	-0.5369	11.4366
	VITA Enamic	7.97925^*^	2.53834	0.005	1.9925	13.9660
IPS e.max CAD	Suprinity	-5.44987	2.53834	0.083	-11.4366	0.5369
	VITA Enamic	2.52938	2.53834	0.580	-3.4574	8.5161
VITA Enamic	VITA Suprinity	-7.97925^*^	2.53834	0.005	-13.9660	-1.9925
	IPS e.max CAD	-2.52938	2.53834	0.580	-8.5161	3.4574

## Discussion

In recent years, endocrown restorations have increased in popularity and have become comparable to conventional crowns in terms of success and survival [11-13]. Dalloul et al. stated that the marginal fit of endocrowns was superior to that of conventional crowns (34.38 µm and 47.08 µm, respectively) [24]. Another study reported marginal gap of 44.66 µm for endocrowns and 73.02 µm for conventional crowns [25].

Based on the results of this study, the null hypothesis that there would be no difference in the marginal gap of endocrown restorations between the three CAD/CAM ceramic materials tested was rejected. Multiple factors can affect the accuracy of the marginal seal and adaptation, such as preparation design [26-28], materials used [29,30], technology advancement, and fabrication methods [31]. Different methods have been used to measure the marginal gap, such as external or internal replica, external or internal microscopic examination [32], and recently, micro-computed tomography (CT) [33]. All previous techniques are reliable methods for marginal gap measurement used in in vivo and in vitro studies [34].

Previous studies have reported the use of destructive methods such as cross-sectioning after cementation, while others have used non-destructive methods such as impression technique, external microscopic examination, and micro-CT [33,34]. In this study, the marginal gap was measured using a digital camera stereomicroscope because it is accurate, reliable, cost-effective, repeatable, and noninvasive. 

For standardization purposes, 30 extracted human mandibular first molar teeth were selected in this study with average dimensions at the level of the cementoenamel junction mesiodistally and buccolingually, and one operator performed the teeth preparations and cementation. Moreover, to reduce human errors, a CAD/CAM scanner and milling units were used to fabricate endocrowns. In addition, for better precision, each of the eight areas selected on each specimen was measured at three different points and their average was calculated.

Previous investigations have reported findings from both marginal and internal (axial, cervical, and pulpal) gap widths of endocrowns. The reported clinically acceptable value for the marginal gap was < 120 µm [35]. This study reported mean gap width values of 26.6 µm for VITA Enamic, 29.1 µm for IPS e.max CAD, and 34.6 for VITA Suprinity. All values were within the reported clinically acceptable range. VITA Suprinity had significantly higher gap width values than VITA Enamic (P=0.005). However, no significant differences in gap width values were found between VITA Enamic and IPS e.max CAD, or between VITA Suprinity and IPS e.max. Previous studies reported superior marginal adaptation of resin materials owing to their lower hardness, modulus of elasticity, and flexural strength; therefore, they have better machinability than ceramic materials [36]. However, other studies have reported that ceramic materials have a smaller marginal gap than resin-based materials. El Ghoul et al. reported marginal gap values of 104.8 for IPS e.max CAD, 114.7 µm for Vita Suprinity, and 143 µm for Cerasmart® (GC International AG, Luzern, Switzerland), and hybrid nanoceramics, respectively [36]. The variation in the findings among these studies could be attributed to different variables, such as the type of restoration, preparation design, restoration material, fabrication techniques, precision, and measurement techniques.

This is an in vitro study in which several variables were more controlled than in vivo studies. Clinically, the scanning process can be challenging because of the presence of saliva and accessibility in the oral cavity. Further studies with larger sample sizes and different gap width measuring techniques are needed to assess the internal and marginal adaptation of endocrowns. 

## Conclusions

The findings of this study concluded that there was a significant difference in the marginal gap of Vita Enamic and Vita Suprinity. However, the reported marginal gap values were in accordance with previous acceptable ranges in the literature. In conclusion, endocrowns fabricated using VITA Enamic, IPS e.max CAD, and VITA Suprinity CAD/CAM materials produced restorations with a clinically acceptable gap width.

## References

[1] Stavropoulou AF, Koidis PT (2007). A systematic review of single crowns on endodontically treated teeth. J Dent.

[2] Suksaphar W, Banomyong D, Jirathanyanatt T, Ngoenwiwatkul Y (2017). Survival rates against fracture of endodontically treated posterior teeth restored with full-coverage crowns or resin composite restorations: a systematic review. Restor Dent Endod.

[3] Tang W, Wu Y, Smales RJ (2010). Identifying and reducing risks for potential fractures in endodontically treated teeth. J Endod.

[4] Gregor L, Bouillaguet S, Onisor I, Ardu S, Krejci I, Rocca GT (2014). Microhardness of light- and dual-polymerizable luting resins polymerized through 7.5-mm-thick endocrowns. J Prosthet Dent.

[5] Sailer I, Makarov NA, Thoma DS, Zwahlen M, Pjetursson BE (2015). All-ceramic or metal-ceramic tooth-supported fixed dental prostheses (FDPs)? A systematic review of the survival and complication rates. Part I: single crowns (SCs). Dent Mater.

[6] Slutzky-Goldberg I, Slutzky H, Gorfil C, Smidt A (2009). Restoration of endodontically treated teeth review and treatment recommendations. Int J Dent.

[7] Dietschi D, Duc O, Krejci I, Sadan A (2007). Biomechanical considerations for the restoration of endodontically treated teeth: a systematic review of the literature--part 1. Composition and micro- and macrostructure alterations. Quintessence Int.

[8] Salehrabi R, Rotstein I (2004). Endodontic treatment outcomes in a large patient population in the USA: an epidemiological study. J Endod.

[9] Ray HA, Trope M (1995). Periapical status of endodontically treated teeth in relation to the technical quality of the root filling and the coronal restoration. Int Endod J.

[10] Gillen BM, Looney SW, Gu LS (2011). Impact of the quality of coronal restoration versus the quality of root canal fillings on success of root canal treatment: a systematic review and meta-analysis. J Endod.

[11] Al-Dabbagh RA (2021). Survival and success of endocrowns: a systematic review and meta-analysis. J Prosthet Dent.

[12] Bindl A, Richter B, Mörmann WH (2006). Survival of ceramic computer-aided design/manufacturing crowns bonded to preparations with reduced macroretention geometry. J Prosthet Dent.

[13] Roggendorf MJ, Kunzi B, Ebert J, Roggendorf HC, Frankenberger R, Reich SM (2012). Seven-year clinical performance of CEREC-2 all-ceramic CAD/CAM restorations placed within deeply destroyed teeth. Clin Oral Investig.

[14] Sofan E, Sofan A, Palaia G, Tenore G, Romeo U, Migliau G (2017). Classification review of dental adhesive systems: from the IV generation to the universal type. Ann Stomatol (Roma).

[15] Spitznagel FA, Boldt J, Gierthmuehlen PC (2018). CAD/CAM ceramic restorative materials for natural teeth. J Dent Res.

[16] Pissis P (1995). Fabrication of a metal-free ceramic restoration utilizing the monobloc technique. Pract Periodontics Aesthet Dent.

[17] Bindl A, Mörmann WH (1999). Clinical evaluation of adhesively placed Cerec endo-crowns after 2 years--preliminary results. J Adhes Dent.

[18] Sedrez-Porto JA, Rosa WL, da Silva AF, Münchow EA, Pereira-Cenci T (2016). Endocrown restorations: a systematic review and meta-analysis. J Dent.

[19] Gresnigt MM, Özcan M, van den Houten ML, Schipper L, Cune MS (2016). Fracture strength, failure type and Weibull characteristics of lithium disilicate and multiphase resin composite endocrowns under axial and lateral forces. Dent Mater.

[20] Belleflamme MM, Geerts SO, Louwette MM, Grenade CF, Vanheusden AJ, Mainjot AK (2017). No post-no core approach to restore severely damaged posterior teeth: an up to 10-year retrospective study of documented endocrown cases. J Dent.

[21] Felton DA, Kanoy BE, Bayne SC, Wirthman GP (1991). Effect of in vivo crown margin discrepancies on periodontal health. J Prosthet Dent.

[22] Amina Amina, Rajput G, Ahmed S (2022). Comparison of microleakage in nanocomposite and amalgam as a crown foundation material luted with different luting cements under CAD-CAM milled metal crowns: an in vitro microscopic study. Polymers (Basel).

[23] Holmes JR, Bayne SC, Holland GA, Sulik WD (1989). Considerations in measurement of marginal fit. J Prosthet Dent.

[24] Dalloul R, Nassar J, Al-Houri N (2016). A comparative study of marginal fit between IPS e.max press crown and endocrown after cementation. Clinic Med and Diagnost.

[25] Abo-Elmaged A, Abdel-Aziz M (2015). Influence of marginal preparation design on microleakage and marginal gap of endocrowns cemented with adhesive resin cement. Egypt Dent J.

[26] Shin Y, Park S, Park JW, Kim KM, Park YB, Roh BD (2017). Evaluation of the marginal and internal discrepancies of CAD-CAM endocrowns with different cavity depths: an in vitro study. J Prosthet Dent.

[27] Gaintantzopoulou MD, El-Damanhoury HM (2016). Effect of preparation depth on the marginal and internal adaptation of computer-aided design/computer-assisted manufacture endocrowns. Oper Dent.

[28] Seo D, Yi Y, Roh B (2009). The effect of preparation designs on the marginal and internal gaps in Cerec3 partial ceramic crowns. J Dent.

[29] Coldea A, Swain MV, Thiel N (2013). In-vitro strength degradation of dental ceramics and novel PICN material by sharp indentation. J Mech Behav Biomed Mater.

[30] Awada A, Nathanson D (2015). Mechanical properties of resin-ceramic CAD/CAM restorative materials. J Prosthet Dent.

[31] Yun MJ, Jeon YC, Jeong CM, Huh JB (2017). Comparison of the fit of cast gold crowns fabricated from the digital and the conventional impression techniques. J Adv Prosthodont.

[32] Abduo J, Lyons K, Swain M (2010). Fit of zirconia fixed partial denture: a systematic review. J Oral Rehabil.

[33] Ekici Z, Kılıçarslan MA, Bilecenoğlu B, Ocak M (2021). Micro-CT evaluation of the marginal and internal fit of crown and inlay restorations fabricated via different digital scanners belonging to the same CAD-CAM system. Int J Prosthodont.

[34] Goujat A, Abouelleil H, Colon P, Jeannin C, Pradelle N, Seux D, Grosgogeat B (2018). Mechanical properties and internal fit of 4 CAD-CAM block materials. J Prosthet Dent.

[35] Boitelle P, Mawussi B, Tapie L, Fromentin O (2014). A systematic review of CAD/CAM fit restoration evaluations. J Oral Rehabil.

[36] El Ghoul WA, Özcan M, Ounsi H, Tohme H, Salameh Z (2020). Effect of different CAD-CAM materials on the marginal and internal adaptation of endocrown restorations: an in vitro study. J Prosthet Dent.

